# Zinc tolerant plant growth promoting bacteria alleviates phytotoxic effects of zinc on maize through zinc immobilization

**DOI:** 10.1038/s41598-020-70846-w

**Published:** 2020-08-17

**Authors:** Devendra Jain, Ramandeep Kour, Ali Asger Bhojiya, Ram Hari Meena, Abhijeet Singh, Santosh Ranjan Mohanty, Deepak Rajpurohit, Kapil Dev Ameta

**Affiliations:** 1 Department of Molecular Biology and Biotechnology, Rajasthan College of Agriculture, Maharana Pratap University of Agriculture and Technology, Udaipur, Rajasthan 313001 India; 2 Department of Soil Science and Agricultural Chemistry, Rajasthan College of Agriculture, Maharana Pratap University of Agriculture and Technology, Udaipur, Rajasthan 313001 India; 3 Department of Biosciences, Manipal University Jaipur, Jaipur, Rajasthan 303007 India; 4 AINP on Soil Biodiversity-Bio-Fertilizers, Indian Institute of Soil Science, Nabibagh, Berasia Road, Bhopal, Madhya Pradesh 462038 India; 5 Department of Horticulture, Rajasthan College of Agriculture, Maharana Pratap University of Agriculture and Technology, Udaipur, Rajasthan 313001 India; 6 Department of Agriculture and Veterinary Sciences, Mewar University, Chittaurgarh, Rajasthan India

**Keywords:** Biotechnology, Microbiology

## Abstract

The increasing heavy metal contamination in agricultural soils has become a serious concern across the globe. The present study envisages developing microbial inoculant approach for agriculture in Zn contaminated soils. Potential zinc tolerant bacteria (ZTB) were isolated from zinc (Zn) contaminated soils of southern Rajasthan, India. Isolates were further screened based on their efficiency towards Zn tolerance and plant growth promoting activities. Four strains viz*.* ZTB15, ZTB24, ZTB28 and ZTB29 exhibited high degree of tolerance to Zn up to 62.5 mM. The Zn accumulation by these bacterial strains was also evidenced by AAS and SEM–EDS studies. Assessment of various plant growth promotion traits viz*.,* IAA, GA_3_, NH_3_, HCN, siderophores, ACC deaminase, phytase production and P, K, Si solubilization studies revealed that these ZTB strains may serve as an efficient plant growth promoter under in vitro conditions. Gluconic acid secreted by ZTB strains owing to mineral solubilization was therefore confirmed using high performance liquid chromatography. A pot experiment under Zn stress conditions was performed using maize (*Zea mays*) variety (FEM-2) as a test crop. Zn toxicity reduced various plant growth parameters; however, inoculation of ZTB strains alleviated the Zn toxicity and enhanced the plant growth parameters. The effects of Zn stress on antioxidant enzyme activities in maize under in vitro conditions were also investigated. An increase in superoxide dismutase, peroxidase, phenylalanine ammonia lyase, catalase and polyphenol oxidase activity was observed on inoculation of ZTB strains. Further, ZIP gene expression studies revealed high expression in the ZIP metal transporter genes which were declined in the ZTB treated maize plantlets. The findings from the present study revealed that ZTB could play an important role in bioremediation in Zn contaminated soils.

## Introduction

Heavy metals are the natural elements having high atomic mass and density (approximately 5 times greater than water)^1^. Heavy metal contamination and exposure is a serious concern for environment and health mainly due to the activities like mining, smelting, use of metals and metal-containing compounds in various applications including agriculture. Many heavy metal ions are essential as trace elements in ppm quantities, but at high concentrations, they turn into toxic elements^2^. These heavy metals are neither remove nor degrade from the environment, unlike the other pollutants that can be degraded by either chemically or biologically means. Excessive levels of heavy metals like zinc, cadmium, copper, lead, nickel and mercury are considered as toxic pollutants^3^.

Elevated concentrations of Zn at toxic levels in the agricultural land from different anthropogenic practices such as application of metal contaminated sewage sludge or from mining activities might represent a potential risk for sustainable and quality food production^4^. In such contaminated soils Zn ions are found at higher concentrations causing toxicity. The applications of plant growth promoting metal tolerant rhizobacteria can be used to decrease such metal toxicity^5^. Considering the significant diversity and capacities of Zn resistance and removal from natural environment, it is essential to identify the candidate microorganisms and also to understand the molecular mechanism of metal removal processes^6^.

Bioremediation is a natural process that uses living organisms or enzymes to detoxify heavy metals from the environment have received great deals of attention^7^. Though the bioremediation approach is relatively slow and time taking, but it is superior over conventional chemical process and most importantly it maintains soil fertility^8^. High concentrations of heavy metals into the environment create selective pressure for the emergence of bacterial strains with tolerance to the metals. These bacteria can affect the reactivity and mobility of such heavy metals and can be used to detoxify some metals preventing further metal contamination^9^.

Zinc (Zn^+2^ cations) cannot diffuse across cell membrane hence specific Zn transporters (Zn-regulated transporter (ZRT), iron-regulated transporter (IRT)-like proteins broadly classified as ZIP protein family) are required for Zn ion homeostasis by regulating Zn uptake and transport^10^. Furthermore, ZIP genes are accountable for the translocation, detoxification and storage of Zn or Fe in the plant cells^11^.

The concentration of Zn over its threshold limit is toxic and reduces plant growth due to reduced photosynthesis, enzyme activity, plant mineral nutrition etc. Hence, heavy metal-tolerant microbes had attended a great deal of interest by researches for Zn bioremediation^12^. Important Zn tolerant PGPR strains include the genus *Cupriavidus*,* Pseudomonas*,* Streptomyces*,* Micrococcus*,* Sphingomonas*,* Klebsiella*,* Serratia*,* Proteus *etc^13–16^. However, there is a need to isolate novel PGPR strains which can perform well in all types of Zn contaminated conditions and importantly their PGP traits should remain active even under Zn stress condition.

The objectives of the present study were to explore the role of ZTB in maize seedlings grown under Zn stress conditions. The effects of these strains were observed upon growth, photosynthetic activities efficiencies and on Zn uptake. Moreover, the role of different Zn transporters genes (ZIPs) involved in Zn uptake and translocation were also analyzed through real time PCR.

## Results

### Analysis of soil samples

In the present study, the rhizospheric soil samples were collected from the Zawar, Udaipur (zinc-lead ore mine tailings areas). The physico-chemical characteristics of the soil samples are described in Table 1. The soil collected in the present study was neutral to slightly alkaline in nature. The rhizospheric soils of Zn contaminated soils contains moderate to high range of EC, OC, total N, total P and total K which might be due to associated PGPRs. The higher Zn contents are attributed to the Zn smelting and mining activities in this area. The diethylene triamine pentacetate acid (DTPA) extractable concentrations of Zn were found to be 35.99 mg/kg and 39.99 mg/kg in Mochia and Balaria mining regions respectively.

**Table 1:** Soil sample sites and chemical properties of experimented soil.

Place	Satellite location	EC^a^ (dS/m)	pH^a^	OC (g/kg)	Av. N (kg/ha)	Av. P (kg/ha)	Av. K (kg/ha)	DTPA-Zn (mg/kg)
Mochia, Zawar	24° 21′ 37.6" N 73° 41′ 45.3" E	0.57	7.19	0.55	94.82	20.20	199.36	35.99
Balaria, Zawar	24° 35′ 38.8" N 73° 75′ 21.1" E	0.62	7.25	0.60	81.09	18.22	169.44	39.99

^a^1:2 soil to water ratio, OC,  organic carbon; Av. N, available nitrogen (Kjeldahl digestion); Av. P, available phosphorus (Olsen’s P_2_O_5_); Av. K, available potassium (ammonium acetate extractable K_2_O).

### Zinc tolerance (MIC) for ZTB strains

The ZTB strains isolated from zinc-lead ore mine tailings areas were subjected to determination of their MIC against Zn and four isolates viz*.,* ZTB15, ZTB24, ZTB28, ZTB29 showed the high MIC value of 63.0 mM Zn in the medium were selected for the characterization.

### ZTB strains characterization

The biochemical characterization of ZTB strains viz*.,* ZTB15, ZTB24, ZTB28 and ZTB29 were summarized in Table 2. Among these 4 strains, all 4 strains were positive for citrate utilization, 3 strains were positive for starch hydrolysis, 1 strain (ZTB24) was positive for nitrate reduction, one strain (ZTB15) was positive for gelatin hydrolysis, all 4 strains were positive for catalase activity and 1 strain (ZTB28) was positive for oxidase activity.

**Table 2:** Biochemical characterization of zinc tolerant bacteria.

Strain name	Starch hydrolysis	Citrate utilization	Nitrate reduction	Gelatin liquefaction	Catalase activity	Oxidase
ZTB 15	**−**	** + **	**−**	** + **	** + **	**−**
ZTB 24	** + **	** + **	** + **	**−**	** + **	**−**
ZTB 28	** + **	** + **	**−**	**−**	** + **	** + **
ZTB 29	** + **	** + **	**−**	**−**	** + **	**−**

** +, Positive; −, negative.**

**Figure 1:**
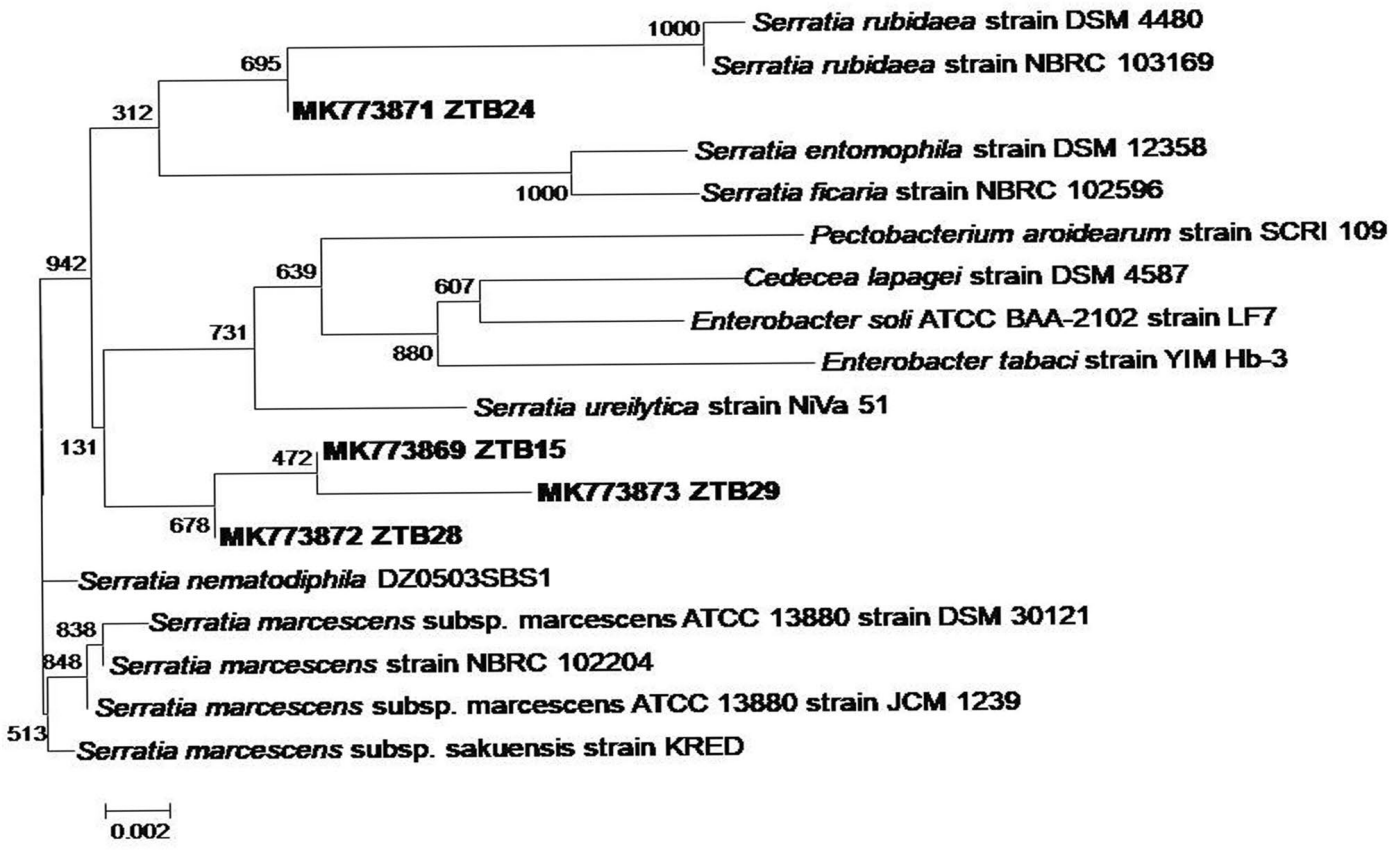
Phylogenetic tree of ZTB strains based on 16S rDNA.

The partial 16S rDNA sequence of ZTB15, ZTB24, ZTB28 and ZTB29 strains were sequenced and analyzed using the BLAST tool. The BLAST results revealed greatest sequence identity of ZTB strains with the previously reported type strains of genus *Serratia*. The phylogenetic position of these ZTB strains is shown in Fig. 1. The NCBI GenBank accession number assigned to the ZTB strains are following:

1. ZTB15: *Serratia* sp. (Accession Number: MK773869)
2. ZTB24: *Serratia* sp. (Accession Number: MK773870)
3. ZTB28: *Serratia* sp. (Accession Number: MK773872)
4. ZTB29: *Serratia* sp. (Accession Number: MK773873)

### Zinc biosorption potential of ZTB strains

The results of Zn biosorption were summarized in Table 3. The results obtained revealed that all the selected ZTB strains were able to remove Zn from the nutrient broth medium efficiently and the highest biosorption of Zn was recorded in the bacterial strain ZTB15 followed by ZTB29, ZTB28 and ZTB24. The results of Zn biosorption were calculated as percentage biosorption. In nutrient broth medium supplemented with concentration of Zn (20 mg/L), ZTB15 was able to remove the highest amount of Zn from the medium i.e. 92.46%. At higher concentration of Zn (40 mg/L), ZTB15 was able to remove 93.51% of Zn from the medium which was the highest among all the ZTB strains. It was closely followed by ZTB28 and ZTB29 with 91.87% and 91.01% respectively of Zn biosorption efficiency. The ZTB strain ZTB24 was able to remove 76.04% of Zn.

**Table 3:** Effect of Zn concentration on biosorption of Zn by ZTB.

Strain name	Concentration of Zn (mg/L) in the supernatant after biosorptionby ZTB after 72 h		% Biosorption of Zn by ZTB after 72 h	
	Media with 20 mg/L Zn	Media with 40 mg/L Zn	Media with 20 mg/L Zn	Media with 40 mg/L Zn
ZTB 15	1.508 ± 0.196^a^	2.598 ± 0.252^a^	92.46	93.51
ZTB 24	3.285 ± 0.020^c^	9.586 ± 0.121^c^	83.58	76.04
ZTB 28	1.831 ± 0.050^b^	3.851 ± 0.059^b^	90.85	91.87
ZTB 29	1.825 ± 0.309^ab^	3.597 ± 0.252^b^	90.88	91.01

Data is presented as means of 3 replicates ± SD (standard deviation). The Mean value followed by same letter in column of each treatment is not significant difference at *p* = 0.05 by Tukey–Kramer HSD test.

### Scanning electron microscopy-energy dispersive X-ray spectroscopy (SEM–EDS) studies of ZTB strains

**Figure 2:**
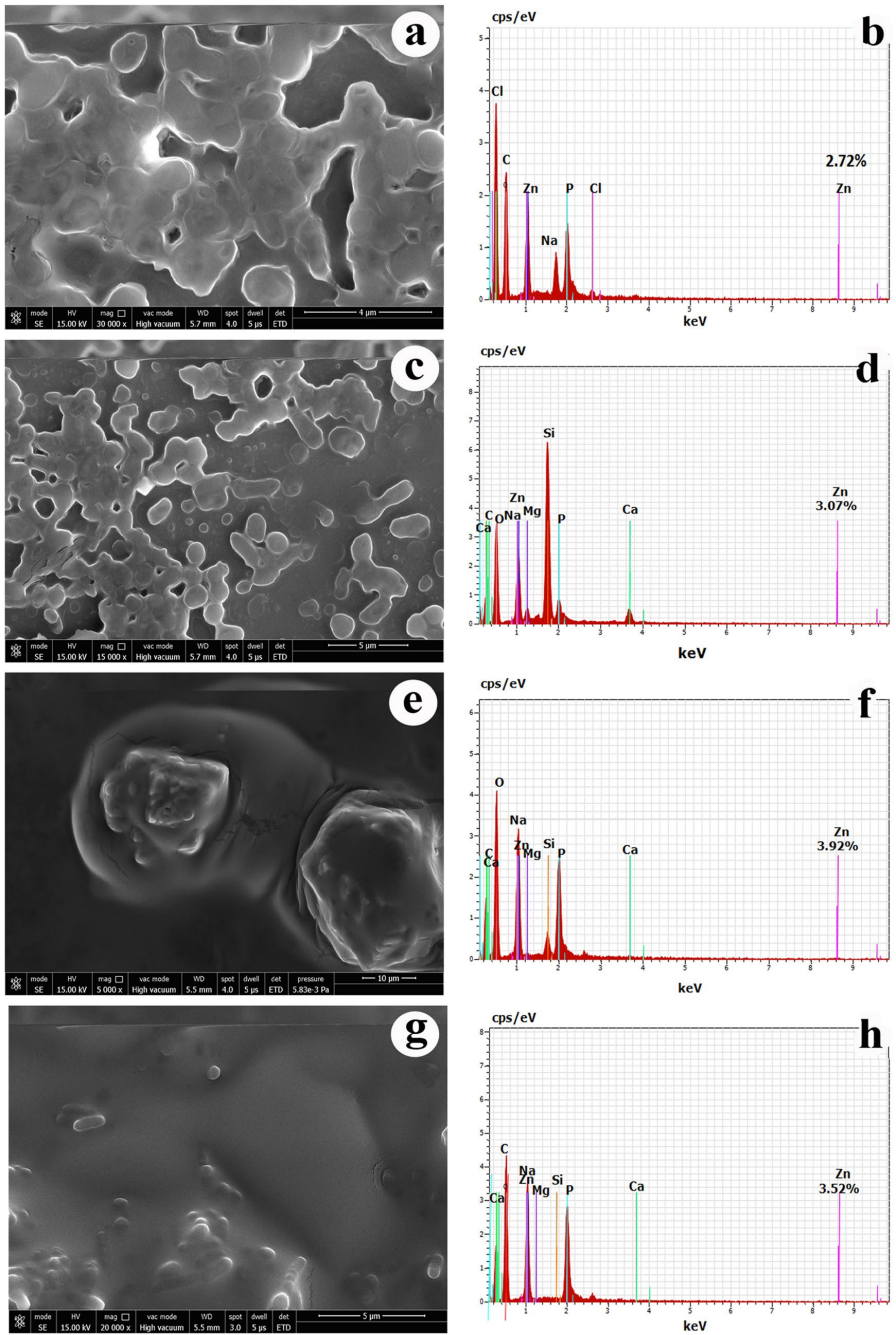
Scanning electron microscopy (SEM) images (**a**) ZTB15, (**c**) ZTB24, (**e**) ZTB28, (**g**) ZTB29 and energy dispersive X-ray (EDX) spectra of (**b**) ZTB15, (**d**) ZTB24, (**f**) ZTB28, (**h**) ZTB29 of Zn treated cells.

Scanning electron microscopy (SEM) was used to examine the morphology of bacterial cells after 72 h exposure to 100 mg/L of Zn whereas the EDS analysis of the bacterium established the presence of elemental content on the microbial biomass (Fig. 2). It was illustrated from the SEM micrographs that the ZTB produces very high amount of exopolysaccharide (EPS) in response to very high concentration of Zn whereas these strains without Zn did not produced EPS (supplementary data sheet). The EDS micrograph of control (heavy metal free) biomass showed only prominent peaks of alkali and alkaline earth metal indicating the presence of these elements in the bacterial biomass (Fig. 2). Further, in the Zn treated bacterial cell samples the peak of Zn metal along with the earlier peaks of the alkali and alkaline earth metal were appeared in the ZTB strains. The EDS analysis showed that the amount of Zn accumulated in the ZTB cells was a maximum of 3.97% and a minimum of 2.72%.

### Plant growth promoting traits of the ZTB strains

All the 4 ZTB isolates were subjected to various plant growth promoting activities viz*.,* IAA production, ACC deaminase activity, siderophore production, phosphate solubilization, potash solubilization, silica solubilization, ammonia production, phytase production and volatile compounds production i.e. HCN production were summarized in Table 4. All the 4 ZTB strains were positive for IAA production and the isolate ZTB29 showed significantly higher IAA production (12.45 µg/mL). All the 4ZTB isolates showed positive results in ACC deaminase activity and ammonia production and showed negative results in production of HCN. All the 4 ZTB strains were positive for GA_3_ production and the isolate ZTB24 produced the highest amount of GA_3_ (60.60 µg/mL). All the 4 ZTB strains were able to solubilize tricalcium phosphate forming holo zones from which phosphate solubilization index (PSI) was calculated. The phosphate solubilization ability of the ZTB15 strain was significantly higher (4.6) in comparison to other three strains. All the 4 ZTB strains were able to solubilize potash and form the clear zones on the medium, the potash solubilization index (KSI) was calculated (supplementary data sheet). The highest KSI was showed by isolate ZTB29 (8.0). The silica solubilization ability of the isolate ZTB28 was significantly higher with silica solubilization index (SSI) of 3.52 cm in comparison to other three strains. All the 4 ZTB strains were found positive for phytase production. The phytase production index (PPI) was highest for isolate ZTB15 (12.12). All the 4 ZTB strains were found positive for siderophore and the siderophore production index was highest for isolate ZTB15 (2.08). Thus presence of these important PGP traits in ZTB strains could provide plant growth promotion and Zn tolerance under Zn stress.

**Table 4:** Plant growth promoting activities of ZTB.

PGPR activity	ZTB strains			
	ZTB15	ZTB24	ZTB28	ZTB29
IAA production (µg/mL)	4.83 ± 0.02	4.32 ± 0.040	8.03 ± 0.02	12.54 ± 0.07
ACC deaminase activity	+ +	+	+ +	+
Ammonia production (µg/mL)	1.42 ± 0.23	1.49 ± 0.56	1.48 ± 0.18	1.45 ± 0.86
HCN production	–	–	–	–
GA_3_ (µg/mL)	28.20 ± 1.31	60.60 ± 1.50	40.86 ± 1.23	28.10 ± 1.01
Phosphate solublization index	4.60 ± 0.10	3.45 ± 0.10	4.10 ± 0.20	3.85 ± 0.04
Potassium solublization index	4.20 ± 0.05	6.30 ± 0.05	6.33 ± 0.03	8.00 ± 0.10
Silica solublization index	2.23 ± 0.02	2.90 ± 0.01	3.52 ± 0.01	2.30 ± 0.01
Phytase production index	12.12 ± 0.01	11.42 ± 0.01	7.50 ± 0.02	11.42 ± 0.01
Siderophore index (Z/C)	2.08 ± 0.01	1.66 ± 0.01	1.11 ± 0.01	2.00 ± 0.60

+, Positive; ++, medium positive; +++, high positive; −, negative; Data is presented as means of 3 replicates ± SD (standard deviation).

### Gluconic acid production

The analysis of ZTB culture supernatant grown under Zn stress conditions allows the detection of gluconic acid produced by ZTB strains. The standard 50% Gluconic acid showed single peak in HPLC chromatogram and was detected at the retention time of 2.22 min (supplementary data sheet). All the four bacterial isolates were able to produce gluconic acid ranging from 293.66 to 382.37 mg/mL. Based on comparison with the standard 50% gluconic acid used in this study, it was found that strain ZTB15, ZTB24, ZTB28 and ZTB29 generated about 293.66, 297.82, 301.13 and 382.37 mg/mL gluconic acid respectively.

### In vitro plant growth promotion by ZTB on maize under Zn stress

The pot culture experiments were conducted under net house conditions in plastic pots filled with sterile planting mixture. The plant growth promoting activities of ZTB isolates under Zn stress conditions were studied on maize plantlets treated with ZTB inoculants (seed bacterization method) under Zn stress conditions (1,000 mg Zn/kg planting mixture). Four ZTB strains viz; ZTB15, ZTB24, ZTB28 and ZTB29 were selected and pot experiment data were recorded under Zn stress condition after 30 days of germination were summarized in Table 5. In uninoculated control (pot containing 1,000 mg Zn/kg planting mixture) the overall all plant growth and chlorophyll content was significantly decreased due to the Zn stress compared to control plantlets (without any Zn stress) (Supplementary Data Sheet). Whereas, higher plant growth and chlorophyll content were observed in maize plantlets treated with ZTB strains compared to uninoculated control. The maize plantlets inoculated with ZTB28 showed best response compare to the other strains and uninoculated control. All the treatments significantly influenced the observed parameters. All the ZTB strains significantly influenced the observed parameters and contributed to plant growth under Zn stress conditions.

**Table 5:** In vitro studies on the effect of zinc tolerant bacteria on growth and biomass of maize seedling under Zn stress conditions (1,000 mg Zn/kg planting mixture).

Treatment details	Average shoot length (cm)	Average root length (cm )	Average root number	Average leaf number	Total chlorophyll (µg/mL)
T1: control without Zn and ZTB inoculation	11.50 ± 0.93^c^	38.50 ± 4.03^b^	10.52 ± 0.98^cd^	6.00 ± 1.0^a^	34.14 ± 4.14^b^
T2: control with Zn and without ZTB inoculation	8.90 ± 1.03^bc^	36.50 ± 3.20^b^	10.13 ± 0.86^d^	5.00 ± 0.58^a^	32.83 ± 4.91^b^
T3: with Zn and ZTB15 inoculation	13.2 ± 1.47^b^	47.23 ± 2.07^a^	13.33 ± 1.32^bc^	5.30 ± 1.15^a^	47.10 ± 4.0^a^
T4: with Zn and ZTB24 inoculation	13.26 ± 1.25^b^	48.56 ± 2.22^a^	14.33 ± 1.25^b^	6.30 ± 0.58^a^	47.10 ± 3.77^a^
T5: with Zn and ZTB28 inoculation	16.59 ± 0.90^a^	52.96 ± 3.04^a^	17.67 ± 1.23^a^	6.67 ± 1.15^a^	57.87 ± 3.99^a^
T6: with Zn and ZTB29 inoculation	13.85 ± 1.10^ab^	50.23 ± 1.94^a^	14.33 ± 1.08^b^	5.30 ± 1.15^a^	48.67 ± 4.26^a^
CD at 5%	2.21	4.42	2.21	2.01	6.73
CV%	14.08	7.97	13.61	28.93	12.44

Data are recorded after 30 days of germination; data is presented as means of 4 replicates ± SD (standard deviation). The Mean value followed by same letter in column of each treatment is not significant difference at *p* = 0.05 by Tukey–Kramer HSD test.

### Antioxidant enzymes activities

The stress related enzymes viz*.,* catalase (CAT), superoxide dismutase (SOD), peroxidase (POD), polyphenol oxidase (PPO) and phenylalanine ammonia lyase (PAL) were also studied in 14 days old seedlings (Table 6). After 14 days of growth under in vitro conditions, the expression of stress related enzymes viz*.,* catalase (CAT), superoxide dismutase (SOD), peroxidase (POD), polyphenol oxidase (PPO) and phenylalanine ammonia lyase (PAL) were significantly lower in control plantlets (without Zn stress) and the plantlet treated with selected ZTB strains compared to the uninoculated control under Zn stress conditions. The maize plantlet treated with selected ZTB isolates showed lower activity of SOD ranged from 0.33 to 0.39 unit/mg fresh weight compare to 0.27 unit/mg fresh weight of uninoculated control under Zn stress conditions and highest activity of SOD were observed in the plantlets treated with ZTB24 whereas lowest activity was observed in plantlets treated with ZTB28.

**Table 6:** In vitro studies on the effect of ZTB on stress related enzymes of maize seedling under Zn stress conditions (1,000 mg Zn /kg planting mixture).

Treatment details	SOD (unit/mg) fresh weight	POD (µmole/min/g)	PAL (µmole/min/g)	Catalase (µmole/min/g)	PPO (µmole/min/g)
T1: control without Zn and ZTB inoculation	0.21 ± 0.02f	1.80 ± 0.18^g^	0.0203 ± 0.002^fg^	18.50 ± 0.41^d^	0.0127 ± 0.001^d^
T2: control with Zn and without ZTB inoculation	0.27 ± 0.02^ab^	1.95 ± 0.30^ab^	0.0213 ± 0.001^ab^	19.23 ± 0.25^a^	0.0141 ± 0.001^c^
T3: with Zn and ZTB15 inoculation	0.36 ± 0.03^cde^	2.82 ± 0.20^bc^	0.0233 ± 0.006^g^	20.92 ± 1.95^cd^	0.0170 ± 0.002^a^
T4: with Zn and ZTB24 inoculation	0.39 ± 0.03^bc^	2.27 ± 0.25^ef^	0.0283 ± 0.002^cdef^	22.58 ± 1.26^c^	0.0163 ± 0.002^b^
T5: with Zn and ZTB28 inoculation	0.33 ± 0.03^def^	2.20 ± 0.25^ef^	0.0314 ± 0.001^bc^	21.17 ± 2.05^cd^	0.0174 ± 0.001^a^
T6: with Zn and ZTB29 inoculation	0.37 ± 0.03^cd^	2.71 ± 0.25^cd^	0.0301 ± 0.005^cd^	26.53 ± 1.51^ab^	0.0176 ± 0.002^a^
CD at 5%	0.050	0.460	0.010	1.430	0.001
CV%	8.61	11.15	14.10	3.65	4.42

*Value is mean of 4 replicates. The Mean value followed by same letter in column of each treatment is not significant difference at *p* = 0.05 by Tukey–Kramer HSD test.

The maize plantlet treated with selected ZTB isolates showed lower activity of POD ranged from 2.20 to 2.82 µmole/min/g compare to 1.95 µmole/min/g of uninoculated control under Zn stress conditions and highest activity of POD were observed in the plantlets treated with ZTB15 whereas lowest activity was observed in plantlets treated with ZTB28. Similarly, the PAL activity were ranged from 0.0233 to 0.0314 µmole/min/g compare to 0.0213 µmole/min/g of uninoculated control under Zn stress conditions and highest amount of PAL produced by ZTB28 whereas lowest amount of PAL produced by ZTB15. The expression of CAT in maize plantlet were ranged from 20.92 to 26.53 µmole/min/g compare to 19.23 µmole/min/g of uninoculated control under Zn stress conditions and highest amount of CAT produced by ZTB29 whereas lowest amount of CAT produced by ZTB15. Whereas, in case of PPO, the ZTB treated plantlets expressed the PPO enzyme ranged from 0.0163 to 0.0176 µmole/min/g compare to 0.0141 µmole/min/g of uninoculated control under Zn stress conditions and highest amount of PPO produced by ZTB29 whereas lowest amount of PPO produced by ZTB24.

### Analysis of Zn uptake in maize seedling using atomic absorption spectroscopy

Accumulation of Zn in the maize plantlet after 30 days of germination under Zn stress conditions (1,000 mg Zn/kg planting mixture) were summarized in Table 7. The results indicated that the treatment with ZTB strains reduced the Zn uptakes in maize seedling significantly compare to the untreated plants. Moreover, inoculated and un-inoculated shoot system exhibited greater Zn accumulation than the roots.

**Table 7:** In vitro studies on the effect of ZTB on Zn accumulation in maize seedling under Zn stress conditions (1,000 mg Zn /kg planting mixture).

Treatment details	Zn concentration in shoot (µg/g fresh weight)	Zn concentration in root (µg/g fresh weight)
T1: control without Zn and ZTB inoculation	65.01 ± 5.0^d^	46.03 ± 6.5^e^
T2: control with Zn and without ZTB inoculation	632.64 ± 6.0^a^	487.90 ± 11.5^a^
T3: with Zn and ZTB15 inoculation	356.28 ± 5.1^b^	299.70 ± 10.1^b^
T4: with Zn and ZTB24 inoculation	335.31 ± 7.6^bc^	280.20 ± 5.5^bc^
T5: with Zn and ZTB28 inoculation	333.12 ± 7.5^c^	262.20 ± 7.0^c^
T6: with Zn and ZTB29 inoculation	339.57 ± 7.1^bc^	218.70 ± 4.45^d^
CD at 5%	2.44	6.22
CV%	0.39	1.29

Each value is mean of 4 replicates. The Mean value followed by same letter in column of each treatment is not significant difference at *p* = 0.05 by Tukey–Kramer HSD test.

### Gene expression analysis

**Figure 3:**
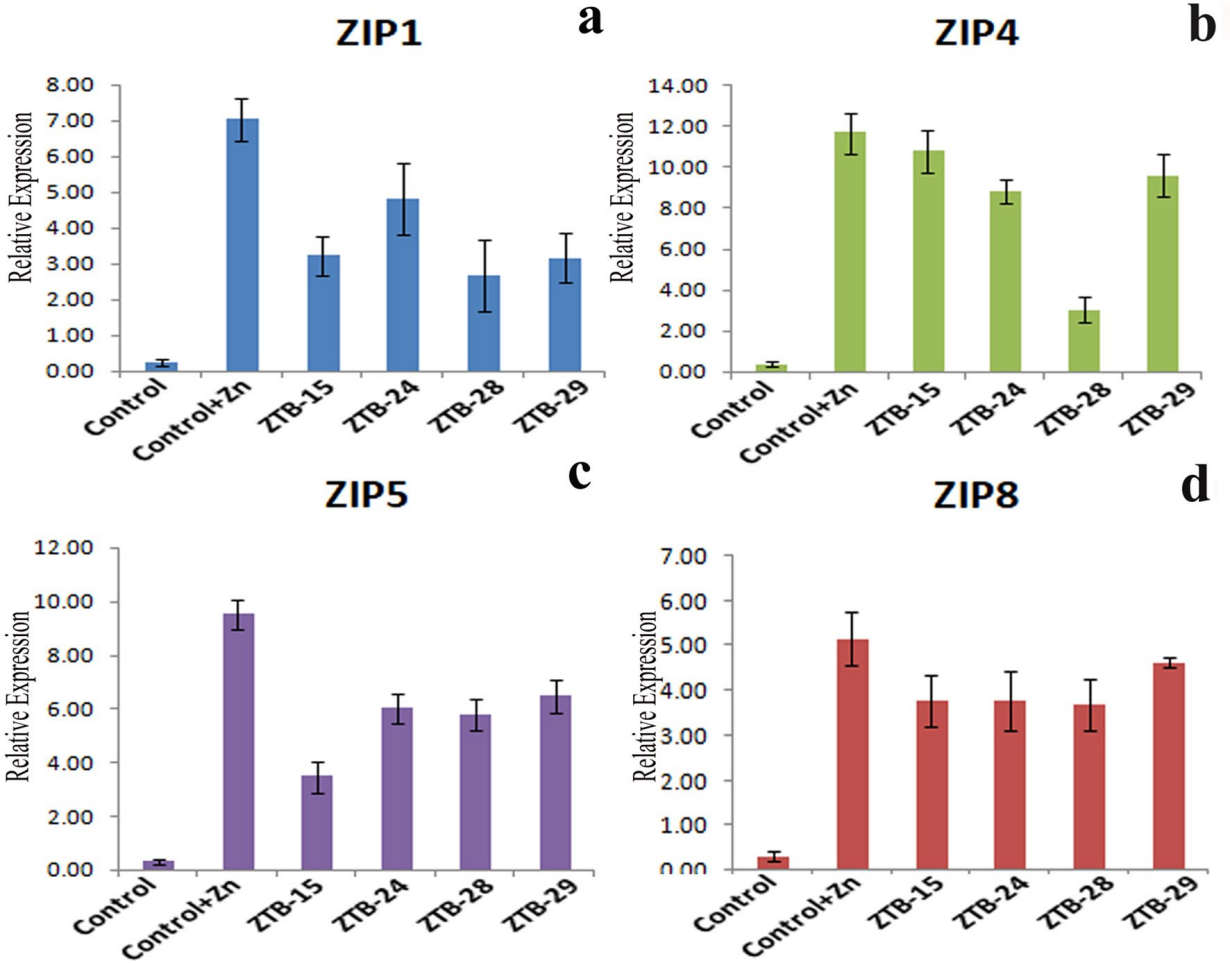
Expression pattern of ZmZIP genes in 14 days maize plantlet under Zn stress conditions (1,000 mg Zn/kg planting mixture) (**A**) ZIP1, (**B**) ZIP4, (**C**) ZIP5 and (**D**) ZIP8 (treatments: control: without Zn and ZTB inoculation; control + Zn: with Zn and without ZTB inoculation; ZTB-15: ZTB-24: ZTB-28: ZTB-29: with Zn and ZTB inoculation).

Zinc treated maize plantlet showed a significant increase in the ZIP metal transporter gene expression under Zn stress as compared to control without Zn stress (Fig. 3). Gene expression studies in 14 days old maize seedlings revealed up regulation of ZIP1, ZIP4, ZIP5 and ZIP8 metal transporter genes in Zn-treated seedlings in response to control without Zn stress. The ZTB inoculation substantially reduced the metal transporter ZIP expression in maize in the presence of Zn.

The seedlings treated with ZTB15 showed decline in the expression of metal transporter ZIP1, ZIP4, ZIP5 and ZIP8 genes by 54.12, 7.80, 63.41 and 43.56% respectively in response to Zn treated seedlings. The seedlings treated with ZTB24 showed decline in the expression of metal transporter ZIP1, ZIP4, ZIP5 and ZIP8 genes by 62.07, 74.09, 36.80 and 31.40% respectively in response to Zn treated seedlings. The seedlings treated with ZTB28 showed decline in the expression of metal transporter ZIP1, ZIP4, ZIP5 and ZIP8 genes by 31.67, 24.44, 39.12 and 15.97% respectively in response to Zn treated seedlings. Similar decline in the expression of metal transporter ZIP1, ZIP4, ZIP5 and ZIP8 genes was recorded by 64.63, 17.92, 31.76 and 39.02% respectively on application of ZTB29 in response to Zn treated seedlings.

## Discussion

Zinc contaminated soil has negative impacts on plants as well as soil microbiome however, such soils are enriched with Zn tolerant PGPR hence, the soil sampling is one of the critical criteria for the isolation of such Zn tolerant PGPRs^17^. Many areas in Rajasthan including the Zawar region are contaminated with the toxic amounts of Zn and other heavy metals due to ore mining and other human activities^18^. The rhizospheric soil samples of the present study site were contaminated with a high degree of Zn metals/metalloids and could serve a better source for isolation of plant growth promoting ZTB strains. The DTPA extractable Zn is considered to be the bio-available Zn and having a concern with respect to the toxicity to the environment and plant uptake and utilization^19^. Yang et al.^20^ studied the soil properties of the different Zn contaminated sites of China and reported that the soil organic matter, available nitrogen and phosphorus were significantly high in rhizosphere soil compare to their bulk soils at Zn contaminated sites reveled the role of associated heavy metal tolerant plant growth promoting rhizospheric bacteria.

Microbial remediation of Zn is due to the several mechanisms viz*.,* biosorption of Zn on the cell surface of microbe through the exopolysaccharide (EPS) secretion, Zn bioaccumulation in the microbe due to the cobalt, zinc and cadmium (CZC) transporter genes and Zn bioprecipitation through the production of sulfide precipitates. The ZTB strains tolerated high Zn concentrations also produced exopolysaccharide (EPS) and the presence of CZC genes were also confirmed through PCR (supplementary data sheet). The resistance by the bacteria to the toxic concentration of Zn is achieved through the two efflux mechanisms mediated by P-type ATPase efflux system and resistance-nodulation-division (RND)-driven transporters system^21^. Haroun et al.^22^ reported the tolerance of same bacterial strains against heavy metals and the highest degree of tolerance was observed with Zn.

The four ZTB trains showed maximum MIC, belongs to genus *Serratia* based on the 16SrDNA analysis. The *Serratia* sp. was previously reported for the heavy metal tolerance and bioremediation. Cristani et al. reported the role of *Serratia marcescens* in toxic metal bioremediation viz*.,* Pb, Cd and Cr from polluted environments^23^. However, the isolation and characterization of *Serratia* sp. in Zn tolerance and bioremediation were not reported earlier and to our best knowledge this is the first detailed report on the application of *Serratia* sp. in Zn bioremediation.

The active mode of Zn accumulation by bacteria is designated as bioaccumulations which varies from organism to organism and mainly depend on to the intrinsic biochemical and structural properties of the bacteria. Results of Zn metal removal studies showed that the 4 ZTB strains remove Zn efficiently from the medium. The capacity of microbes to remove toxic heavy metals from growth medium is significantly influenced by growth conditions. Benmalek and Fardeau reported the Zn biosorption efficiency of *Micrococcus* spp. is 59.55 to 78.90% when 25–100 mg/L of Zn was added in the medium^24^. In the present study, similar findings were reported and the Zn accumulation capacities of ZTB were significantly high.

The most possible reasons behind such high heavy metal resistance are due to the phenomena of either bioaccumulation or biosorption. Bioaccumulation of Zn by ZTB strains was evident in AAS studies. The ZTB strains were found to produce significant amount of EPS under Zn stress. EPS mediated Zn biosorption mainly occurs due to the interaction between positively charged Zn ions and negatively charged EPS on the cell surfaces^25^. The present finding was supported by the earlier workers^26,27^ and also confirmed about the ability of alleviating heavy metal stress by microbes.

All ZTB isolates were subjected to various plant growth promoting activities and exhibited multiple PGP traits in vitro which are similar to the earlier findings^28^. Plant growth promoting rhizobacteria positively alters plant growth and its productivity by the production of growth regulators viz*.*, IAA, GA_3_, siderophore etc. which increase the nutrient availability to plants^29^. Compared to other published reports, ZTB strain of the present study possess maximum PGPR activities so far reported and could be helpful for bioremediation in Zn contaminated agricultural land near Zn mining areas.

Gluconic acid is produced in the periplasm and secreted outside the bacterial cells hence can be studied in the supernatant^30^. In the present study all the ZTB strains were able to produce high amount of gluconic acid which non-specifically solubilize Zn, phosphorus, potassium, calcium, manganese etc. from their respective minerals^31^ and moreover may chelate toxic metals resulting in the formation of metallo-organic molecules^32^.

The effects of ZTB strains on the growth of maize plants under Zn^2+^ stress were studied. Under Zn^2+^ stress, a substantial reduction in shoot length, root length, fresh weight, and total chlorophyll was noticed, however inoculation with ZTB strains resulted in significant enhancement in all the plant growth parameters. Zn stresses (1,000 mg Zn/kg planting mixture) resulted in reduced maize plant growth and also induce oxidative damage^33^. The inoculation of plant growth promoting heavy metal tolerant bacteria reduces the metal toxicity and also improves the nutritional status in plants by complex unknown mechanism^29^. Islam et al. reported the inoculation of maize with PGP *Proteus mirabilis* could reduce the negative consequences of oxidative stress caused by heavy metal toxicity^34^.

Crop plants employ detoxifying antioxidative system to maintain ROS at an optimum level. The exposure to the toxic concentrations of heavy metal causes ROS production resulting in high oxidative damage to the crop plant. The antioxidant enzyme i.e. SOD, POD, PAL, PPO and CAT activities in heavy metal stressed plants are depending on the concentration and type of heavy metal, plant species, exposure etc. and most of the cases relatively high compare to control conditions^35^. The higher antioxidant enzyme activity with ZTB strains inoculation in the present study might be due to the increased expression of plant antioxidant enzymes compare to un-inoculated plants. These finding were very well supported by the previously published research where the bacterial inoculation activates the gene expression profile of metal detoxifying enzymes to cope up the metal stress^6,26,27,36,37^.

The improved growth of maize plantlet under Zn stress conditions was due to the reduced accumulation and uptake of Zn in the maize plantlet by ZTB inoculation led to the reduced Zn toxicity. This could happen due to the reduced bioavailability and bioaccumulation of Zn by ZTB strains. Similar findings of metal tolerant PGPR inoculation were found very effective upon inoculation in different crops and also conferred metal tolerance^38^.

The ZIP family transporter genes are responsible for Zn uptake from soil by roots, translocation within root system and from root to shoot and also storage of Zn in various plant parts such as fruits, grains etc. Importantly the relative ZIP gene expression varied between shoot and root^11^. The finding of the quantitative real-time reverse transcription PCR of the ZIP genes from the present study was very well supported by the findings of Khanna et al.^6^. They reported that the enhanced expression of the different metal transporter genes which were further declined in metal tolerant PGPR supplemented plantlets. Hence, the metal tolerant PGPR reduces the heavy metal toxicity and improve the growth of plants on metal contaminated sites.

## Conclusion

The current study was framed to explore zinc tolerant bacteria (ZTB) for improving plant growth under Zn toxicity and define the mechanistic processes regulating the Zn tolerance. Four potential bacterial strains were isolated from a Zn contaminated agricultural field and were identified as *Serratia* sp. In addition to their high tolerance to Zn, these strains also exhibited various plant growth promoting activities. Moreover, experimental evidences also suggested that ZTB strains produced gluconic acid as natural chelating agents of heavy metals and forms metal complexes. To evaluate the extent of PGPR attributes under Zn toxicity rendered by the ZTB, maize plants were inoculated with the strains. Decreased growth of Zn stressed maize plantlet was possibly attributable to the activation of plant defense mechanism and also to the reduced synthesis of plant growth promoting substances. However, the application of ZTB strains significantly improved the growth, antioxidant enzymes activities and decreased the accumulation of Zn in maize plantlet under Zn stress conditions. The results indicated that the ZTB can be used as microbial inoculants for improving agriculture in Zn contaminated soil and bioremediation of heavy metals in polluted industrial sites. Further to confirm the efficacy of these ZTB, dedicated field studies are required on different crops under Zn stress conditions for the determination of Zn bioremediation potential of these isolates.

## Methods

### Soil samples and physico-chemical properties

Rhizospheric soil samples were collected from the Zawar mines areas of Udaipur regions of Southern Rajasthan, India. The collected soil samples were air dried, sieved, and kept for the analysis. Physical and chemical properties of collected soil samples like electrical conductivity (EC), pH, organic carbon (OC) and soil nutrients viz*.,* available nitrogen (Av. N), available phosphorus (Av. P), and available potassium (Av. K) and DTPA extracted Zn were analyzed^39–41^.

### Isolation of zinc tolerant bacteria and determination of minimum inhibitory concentration (MIC) of zinc

The Zn tolerant bacteria were isolated by serial dilution and pour plate methods using nutrient agar amended with 1 mM concentration of zinc sulphate heptahydrate. The plates were incubated at 30 °C for 24 h. The MIC of heavy metals at which no colony growth occurred was determined by the agar dilution method^42^. All the ZTB isolates were grown on nutrient agar plates with gradually increasing the concentration of the Zn ions. The lowest concentration of Zn ions that inhibited the growth of ZTB was taken as the MIC of that metal.

### Characterization of the isolates

Morphological and biochemical identification tests of the ZTB were carried out by using the standard protocol outlined in Bergey’s Manual of Systemic Bacteriology. Molecular identification of the ZTB was done using 16S rDNA amplification and sequencing. The universal primers for 16S r DNA viz*.* 27 F (5′-AGAGTTTGATCMTGGCTCAG-3′) and 1,492 R (3′-TACGGYTACCTTGTTACGACTT-5′) were used for amplification. The amplified PCR product was further purified gel extraction kit (Sigma) and sequenced directly in an automated DNA Sequencer (ABI Prism 310 Genetic Analyzer, Applied Biosystems, Inc., Foster City, CA). This was followed by assembling the sequences by BioEdit software package^43^. Phylogenetic analysis was performed using the obtained aligned sequence followed by the BLAST with the 16S ribosomal RNA sequence (Bacteria and Archaea) nucleotide database. The closest species related to the sequence were retrieved and analyzed by MEGA software 6.0^44^. The neighbor joining method was employed with bootstrap values generated from 1,000 replicates for construction of phylogenetic tree.

### Determination of biosorption potential of zinc tolerant isolates

Biosorption potential of ZTB strains showing higher MIC was determined by atomic absorption spectroscopy (AAS) as the amount of metal present in the supernatant after the treatment with ZTB strains^45^. Nutrient broth supplemented with Zn was inoculated with the 1% of overnight grown ZTB isolate and incubated for 72 h in a shaking condition. The cell free supernatants were used to determine the concentration of Zn biosorption using AAS. The biomass of ZTB strains were also recorded after 72 h biosorption and summarized in (supplementary data sheet).

### Scanning electron microscopy-energy dispersive spectroscopy (SEM–EDS)

The pelleted ZTB cells after biosorption were fixed with 3% glutaraldehyde and further dried under freeze drier (Labtech, India) and placed on the stud surface, there after sputtered with gold particles and imaged with SEM (Carl Zeiss, EVO 181, Germany) operating at 30.0 kV. Further, EDS (Inca Penta FETx3 energy dispersive X-ray system, UK) were also performed to analyses the elemental composition of the surface of ZTB strains after Zn biosorption.

### Screening for multiple plant growth promoting activities

All the selected ZTB inoculates was screened for multiple plant growth promoting activities viz*.,* IAA, ammonia, HCN, Siderophore production and solubilization of phosphorus, potassium and silica standard procedures^46^. The ACC deaminase activity was quantitatively analyzed based on their ability to use ACC as a sole nitrogen source^47^. Production of GA_3_ was carried out using the standard procedure of Berryos et al.^48^. The phytase producing ability of the ZTB were tested on phytase screening medium (PSM) described by Kerovuo et al.^49^.

### Gluconic acid production

The ZTB were tested for the production of gluconic acid by using the injecting the culture filtrate of the isolates in to a HPLC system (Waters, Austria) on a reverse-phase C18 column (Nucleosil 100-5 C18, 250 × 4.6 mm, 5 μm). Elution was performed with an isocratic flow consisting of acetonitrile: water (30:70 v/v) with a flow rate of 1.0 mL/min at 210 nm using UV/Vis detector^50^.

### In vitro studies on the effect of zinc tolerant bacteria on growth and biomass of maize seedling under Zn stress conditions

The pot experiment for selected ZTB isolates showing high MIC values was conducted in plastic pots filled with sterile coco peat/vermiculite/perlite mixture (0.5 kg pot^−1^). Maize cultivable variety (FEM-2) recommended for this agro climatic zone seeds will be used for the in vitro studies. The seeds treated with bacterial inoculant was sown under Zn stress conditions, whereas the uninoculated control under Zn stress condition will also be maintained. The Zn^2+^ concentration in the pots was maintained to 1,000 mg Zn/kg planting mixture. The experimental setup was designed as per previous studies by Gupta et al.^37^. The pots containing Zn contaminated soil were left for two weeks for stabilization of Zn. Six treatments were set up in quadruplicate in a complete randomized design comprising one plant per pot. The details of the treatments are: T1 (control, uncontaminated soil), T2 (soil containing 1,000 mg Zn/kg planting mixture), T3 (soil containing 1,000 mg Zn/kg planting mixture + ZTB15), T4 (soil containing 1,000 mg Zn/kg planting mixture + ZTB24), T5 (soil containing 1,000 mg Zn/kg planting mixture + ZTB28), T6 soil containing 1,000 mg Zn/kg planting mixture + ZTB29). The pots were watered once in 2 days with sterile distilled water until the completion of the study. The maize seed was surface sterilized using 70% ethanol followed by 3% hypochlorite solution for 3 min and used for germination in pot experiment. The concentration of Zn in the pots will be maintained as 1,000 mg Zn/kg planting mixture and the pots will be left for 2 weeks for metal stabilization. The pots will be set in triplicate in a complete randomized design. Different plant growth parameters like average shoot length, root length, root number, leaf number, chlorophyll content of leaf number will be analyzed. The stress related enzymes viz*.,* catalase (CAT), superoxide dismutase (SOD), peroxidase (POD), polyphenol oxidase (PPO) and phenylalanine ammonia lyase (PAL) will be studies in 14 days old seedlings as per the standard protocols previously published^51^. Analysis of Zn content in the maize plantlet was analyzed using atomic absorption spectroscopy (AAS). Briefly 1.0 g of plant material was digested in di-acid mixture containing concentrated nitric acid and concentrated sulphuric acid at 70 °C followed by diluting the samples in distilled water. The extract was filtered and analyzed for total Zn content using AAS.

### Gene expression studies using real-time RT PCR

The 14 day old maize plantlets grown under different treatments were utilized for gene expression studies of different ZIP transporter genes using quantitative real time RT-PCR (Bio-Rad “CFX96 Real-Time PCR System”). The primer details are given in Table 8. Total RNA was isolated from 14 days old maize plantlet with TRIzol (Invitrogen). The cDNA synthesis was done from 5 μg of total RNA as a template using iScript cDNA Synthesis Kit (Bio Red, USA). Real-time RT-PCR was performed in a 20 μL reaction containing a 5 μL cDNA,0.4 μM of gene-specific primers and 10 μL of 2X SYBR Green JumpStart Taq ReadyMix (Sigma). The data was assessed in triplicates and *actin* gene was used as house-keeping control gene for normalization purposes. The data was calculated using threshold cycle (Ct) of the amplification curve. The relative gene expression level was assessed using the 2−ΔΔct method^11^. The sizes of the amplified fragments were confirmed by gel electrophoresis.

**Table 8:** Primers for real-time RT-PCR^10^.

Primer names	Primer sequences
RTZmZIP1F	5′-CCTCTCTGCGTTGGTTGCTCT-3′
RTZmZIP1R	5′-TTGATGGTTGTTTTCTGGTCGT-3′
RTZmZIP4F	5′-CCTTCTTCTCGCTCACCGCT-3′
RTZmZIP4R	5′-AGCCTCGGGTTGCTGAAGT-3′
RTZmZIP5F	5′-GCACATAGGCATAGCCACGC-3′
RTZmZIP5R	5′-ACGCCCAAAGATAGCCCGAT-3′
RTZmZIP8F	5′-CGTGTCATCGCTCAGGTTCTTG-3′
RTZmZIP8R	5′-CCCTCGAACATTTGGTGGAAG-3′
ZmActin1F	5′-ATGTTTCCTGGGATTGCCGAT-3′
ZmActin1R	5′-CCAGTTTCGTCATACTCTCCCTTG-3′

### Statistical analyses

All the observations recorded were subjected to the statistical analysis viz*.* standard deviation (SD), critical difference (CD), coefficient of variation (CV), etc. using Microsoft Excel 2003. The significant difference among variable treatment were determined by the analysis performed in JMP software^52^ version 11 using Turkey–Kramer HSD test at *p* = 0.05.

## Supplementary information

Supplementary Information.

## Abbreviations

Zinc: Zn
ZTB: Zinc tolerant bacteria
PGP: Plant growth promoting
AAS: Atomic absorption spectroscopy
SEM: Scanning electron microscopy
