# Supplementary material for: Zinc tolerant plant growth promoting bacteria alleviates phytotoxic effects of zinc on maize through zinc immobilization
Source: Sci Rep. 2020 Aug 17;10:13865. doi: 10.1038/s41598-020-70846-w (PMC7431563; doi:10.1038/s41598-020-70846-w)
Supplement: Supplementary file 1 — Supplementary Information. [file 41598_2020_70846_MOESM1_ESM.docx]

**Supplementary Data Sheet**

**Zinc tolerant plant growth promoting bacteria alleviates phytotoxic effects of zinc on maize through zinc immobilization**

Figure S1: HPLC Chromatogram of standard gluconic acid (1235 mg/ml) peak detected at RT 2.220


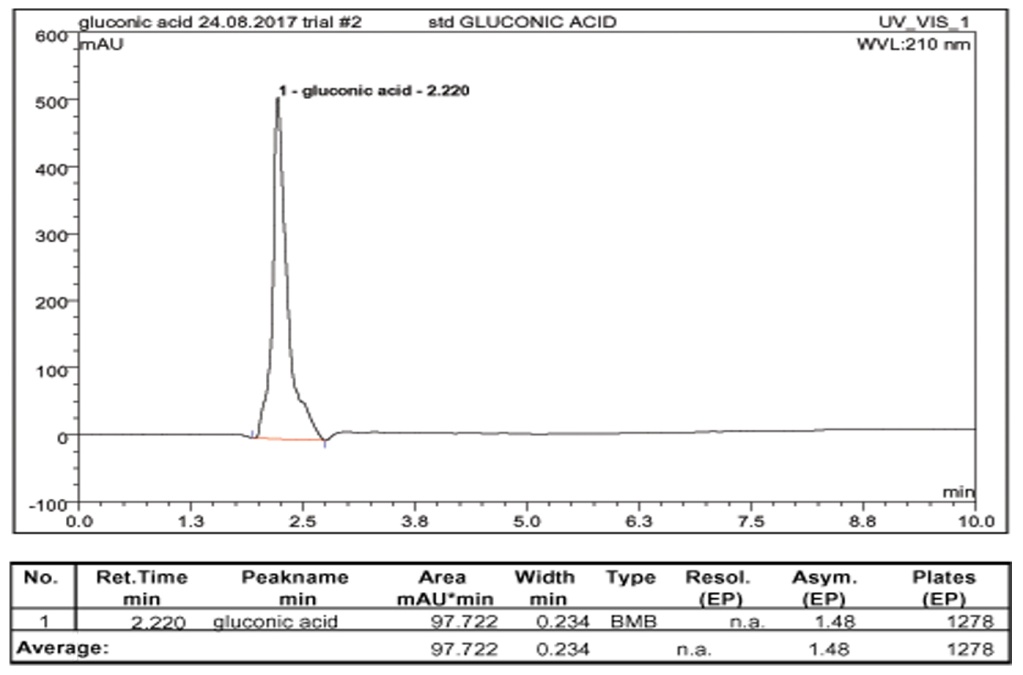


Figure S2: PCR Amplification of partial *czcD* gene in ZTB strains (Primer details CzcD F 5’CAGGTCACTGACACGACCAT 3’ and CzcD R 5’CATGCTGATGAGATTGATGATC 3’; amplicons size: 389 bp; M: 100bp ladder; 1: ZTB15; 2: ZTB24; 3: ZTB28; 4: ZTB29)


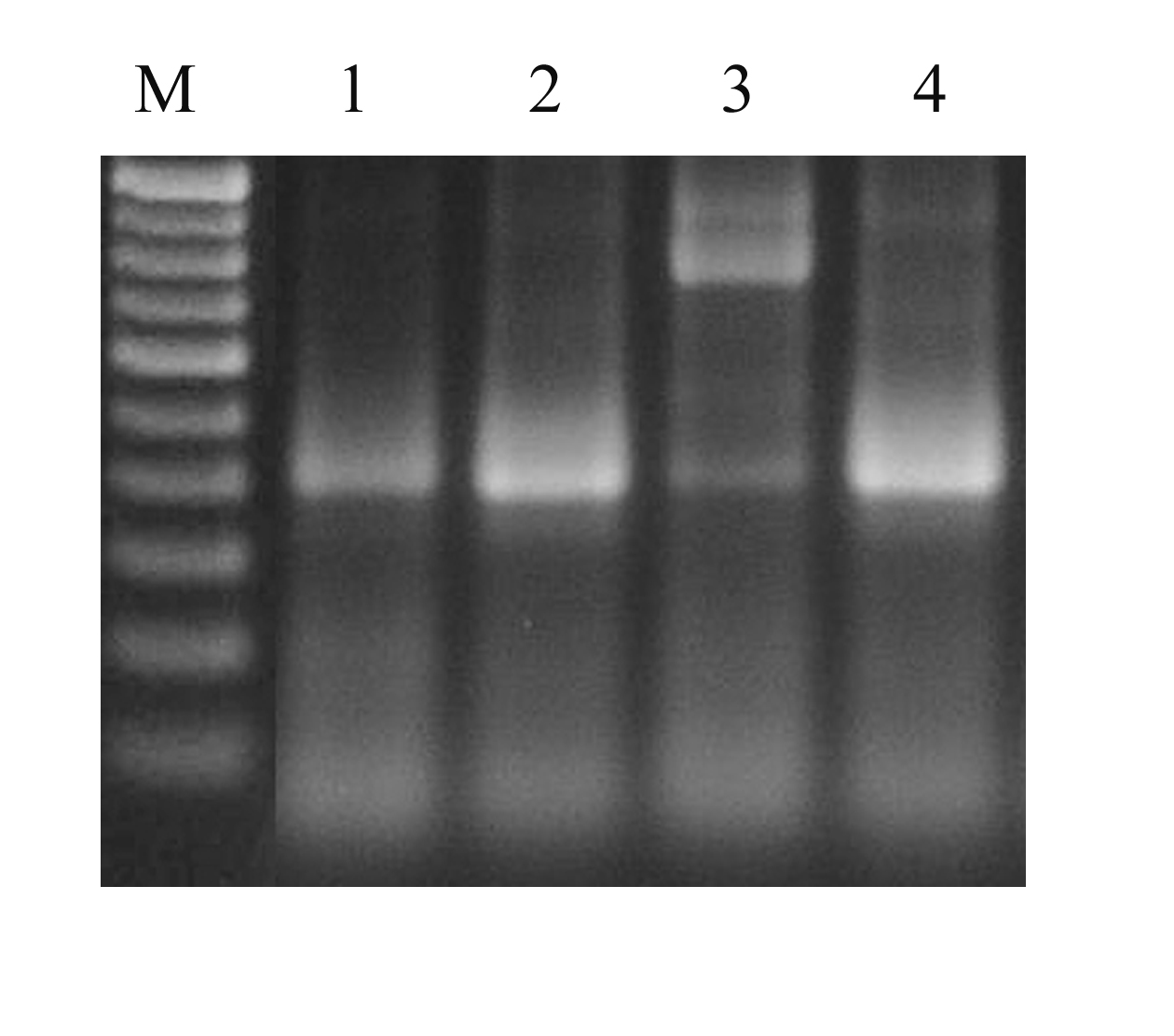


Figure S3: Potassium solubilization activities of ZTB on MSM supplemented with MICA


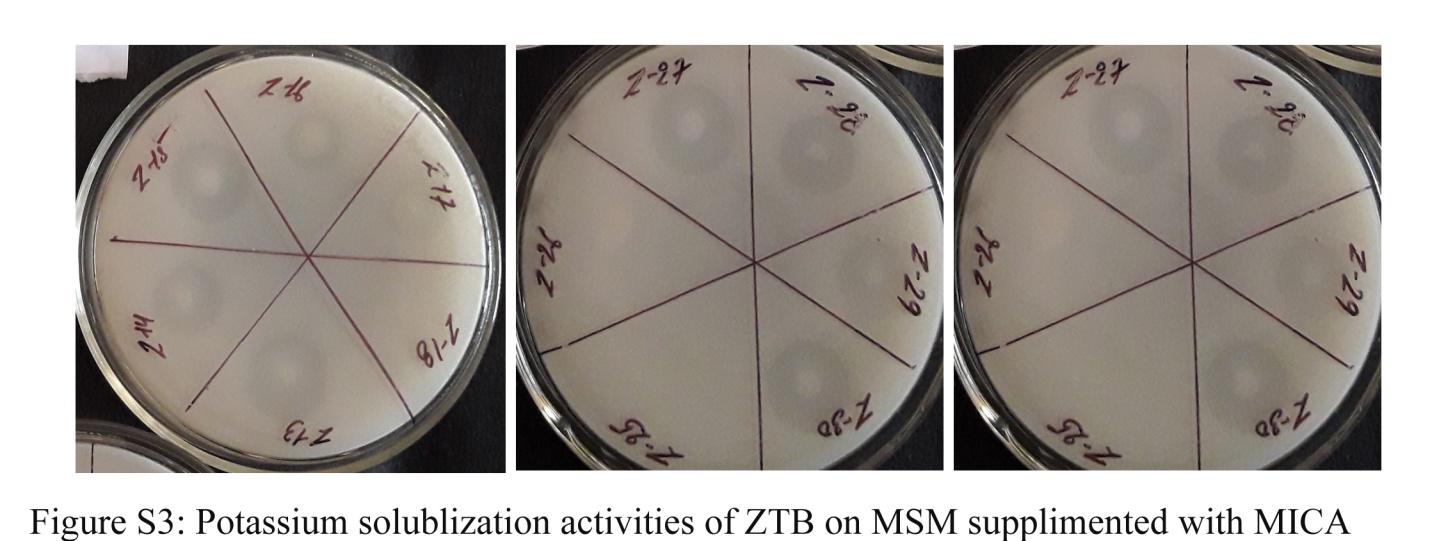


Figure S4: SEM morphology analysis of Zinc tolerant Bacteria (ZTB) without Zn stress

(a) ZTB15 (b) ZTB24 (c) ZTB28 (d) ZTB29

.
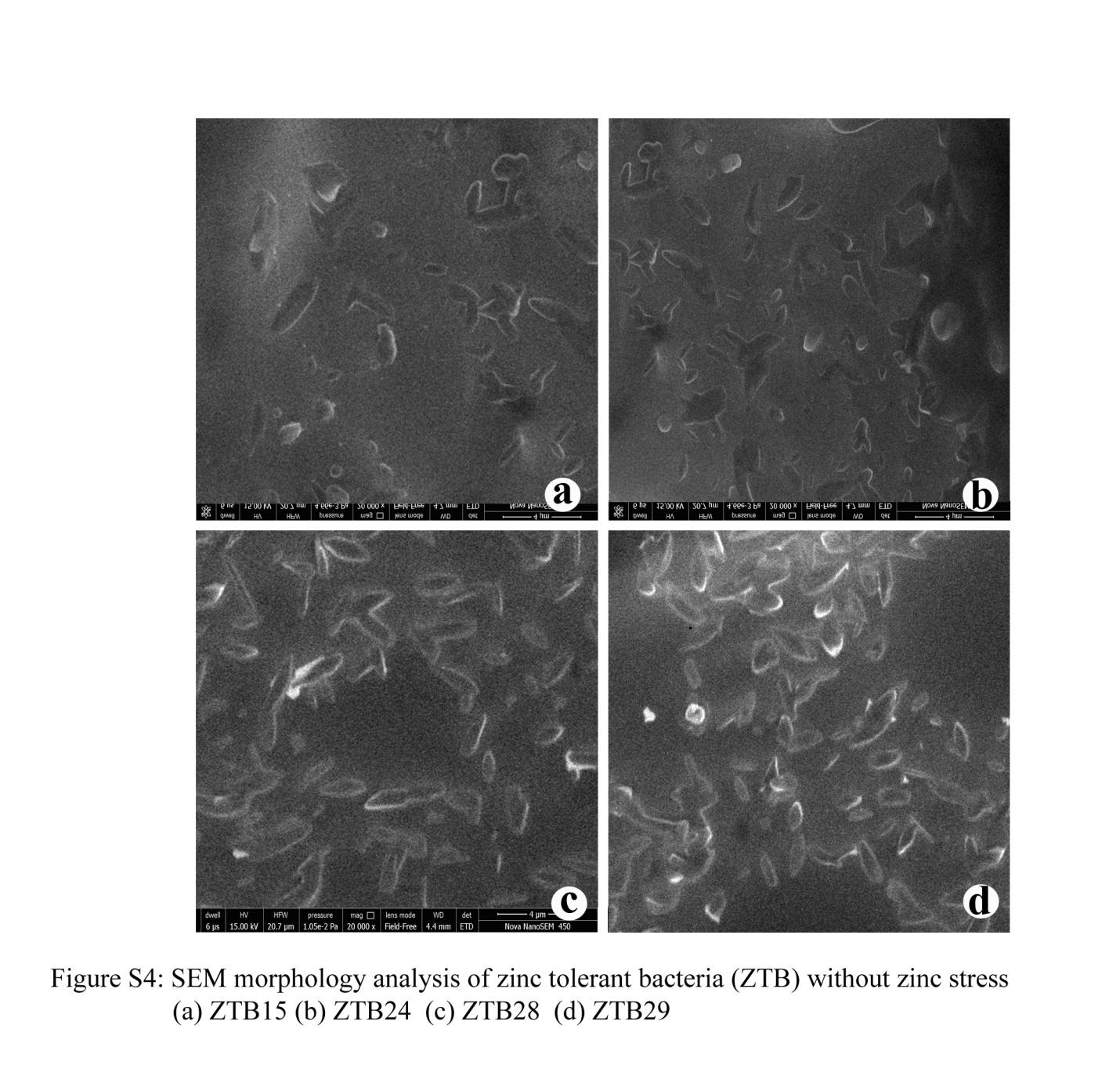


**Figure S5:** *In vitro* studies on the effect of ZTB strains on growth and biomass of maize seedling under zinc stress conditions (1000 mg/kg Zn^2+^) after 30 days of germination

**
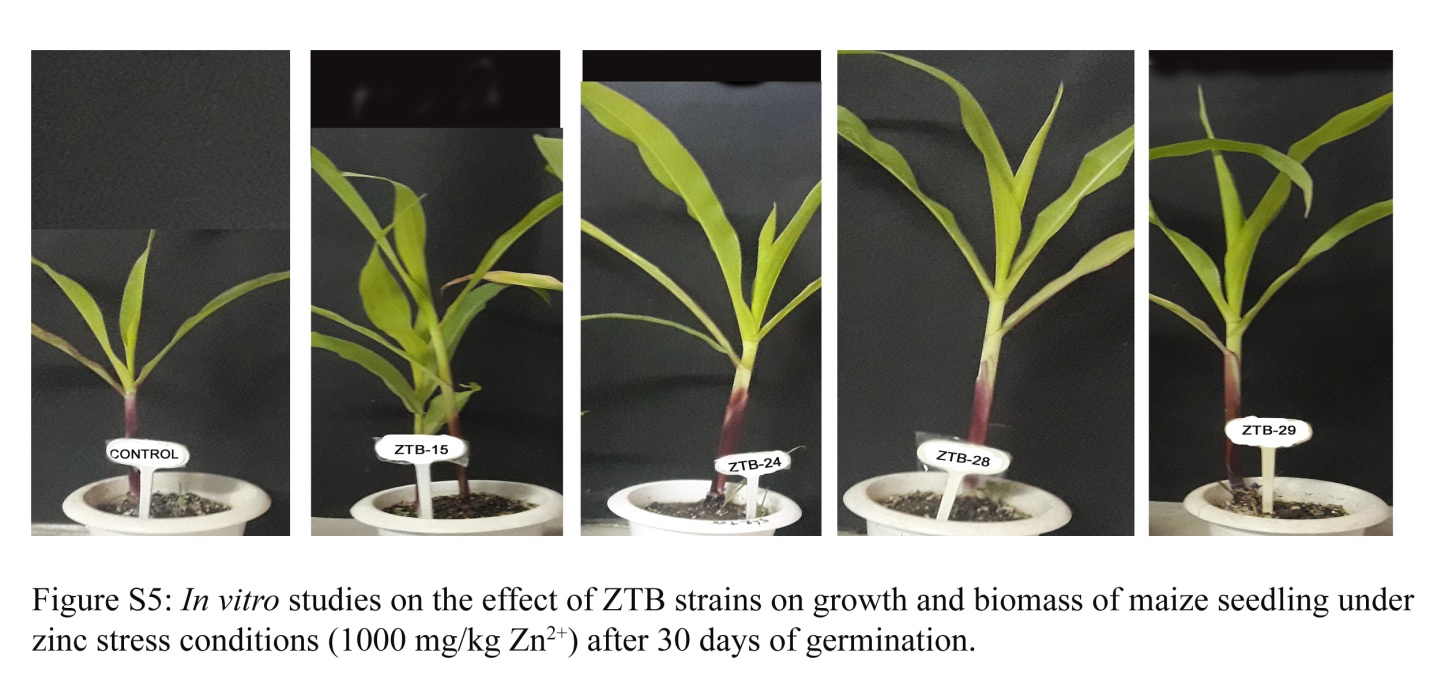
**

**Table S1: Biomass of ZTB strains after Zn biosorption from the media.**

| **Strain Name** | **ZTB Biomass (g/L) after Zn biosorption*** | |
| --- | --- | --- |
|  | **Media with 20 mg/L Zn** | **Media with 40 mg/L Zn** |
| ZTB 15 | 4.73 ± 0.06 | 4.95 ± 0.05 |
| ZTB 24 | 4.43 ± 0.06 | 4.56 ± 0.08 |
| ZTB 28 | 4.61 ± 0.03 | 4.72 ± 0.03 |
| ZTB 29 | 4.65 ± 0.05 | 4.75 ± 0.05 |

Data is presented as means of 3 replicates ± S.D (standard deviation).

* After 72 h of incubation, the ZTB was centrifuged and the pellet obtained were dried at 80°C for 3 h and weighed to obtain final dried biomass.

**Author Contributions-**

Devendra Jain designed the research.

Devendra Jain, Ramandeep Kour, KD Ameta and Ali A Bhojiya performed the experiments and interpreted the data.

RH Meena and Deepak Rajpurohit performed soil and AAS analysis.

Abhijeet Singh performed HPLC and SEM studies.

Devendra Jain, Ali A Bhojiya and SR Mohanty wrote the manuscript.
